# Design of I-WP Gradient Metamaterial Broadband Electromagnetic Absorber Based on Additive Manufacturing

**DOI:** 10.3390/polym17141990

**Published:** 2025-07-20

**Authors:** Yi Qin, Yuchuan Kang, He Liu, Jianbin Feng, Jianxin Qiao

**Affiliations:** 1 School of Mechatronics Engineering, Harbin Institute of Technology, Harbin 150001, China; qy01111@sina.com (Y.Q.); 24s108172@stu.hit.edu.cn (Y.K.); 2 Beijing Institute of Astronautical System Engineering, Beijing 100076, China; 3 Research Institute of Petroleum Exploration & Development, Beijing 100083, China; liuhe@petrochina.com.cn

**Keywords:** lattice structure, electromagnetic wave absorption, additive manufacturing

## Abstract

The proliferation of electromagnetic wave applications has accentuated electromagnetic pollution concerns, highlighting the critical importance of electromagnetic wave absorbers (EMA). This study proposes innovative I-Wrapped Package Lattice electromagnetic wave absorbers (IWP–EMA) based on the triply periodic minimal surface (TPMS) lattice structure. Through a rational design of porous gradient structures, broadband wave absorption was achieved while maintaining lightweight characteristics and mechanical robustness. The optimized three-dimensional configuration features a 20 mm thick gradient structure with a progressive relative density transition from 10% to 30%. Under normal incidence conditions, this gradient IWP–EMA basically achieves broadband absorption with a reflection loss below −10 dB across the 2–40 GHz frequency band, with absorption peaks below −19 dB, demonstrating good impedance-matching characteristics. Additionally, due to the complex interactions of electromagnetic waves within the structure, the proposed IWP–EMA achieves a wide-angle absorption range of 70° under Transverse Electric (TE) polarization and 70° under Transverse Magnetic (TM) polarization. The synergistic integration of the TPMS design and additive manufacturing technology employed in this study significantly expands the design space and application potential of electromagnetic absorption structures.

## 1. Introduction

The rapid advancement of digital technologies and communication systems has not only facilitated the widespread adoption of microwave-based electronic systems but has also accelerated the evolution of intelligent industrial automation [1]. However, the resulting electromagnetic pollution has emerged as a critical threat to human health, equipment reliability, and national defense security [2,3,4,5]. Consequently, the development of thin, lightweight, high-efficiency, and broadband wave-absorbing materials has become a pivotal research focus, playing an indispensable role in electromagnetic protection and stealth technology applications [6,7].

Conventional electromagnetic wave-absorbing structures, primarily utilizing dielectric/magnetic loss materials (e.g., carbon black, graphene, flaky carbonyl iron particles, etc.), have exhibited remarkable absorption performance [8,9]. However, conventional approaches relying on material mixing, microstructural design, and component proportioning constrain the operational bandwidth, failing to meet the multiband absorption requirements. In addition, with the increasing demand for structural and functional integration, the demand for lightweight, high-strength, and impact resistance in practical applications further limits its large-scale application [10,11,12,13,14,15].

Recent advances in additive manufacturing have substantially expanded the design space, enabling multi-scale loss mechanism integration through coordinated material structure designs, which offer novel solutions for electromagnetic wave absorption. As demonstrated by Sun et al., a topology-optimized octet–truss gradient architecture made of carbon black–polylactic acid composites enables electromagnetic absorbers with superior broadband performance [16]. Wei et al. successfully achieved an electromagnetic wave-absorption rate of over 90% in the range of 3.53–24.00 GHz by designing and manufacturing a three-dimensional metamaterial absorber based on a 3D-printed honeycomb structure and resistive film, and demonstrated a high specific strength and energy absorption capability in its mechanical properties [17]. On this basis, Duan et al. further utilized gradient design to fabricate a composite microwave-absorbing element structure composed of flaky carbonyl iron particles and polyether ether ketone composite materials, achieving a −10 dB absorption bandwidth from 5.1 to 40 GHz. In addition, the structure also has incident insensitivity and good mechanical stability [18]. These studies reveal that a lattice structure design enables precise dielectric property modulation, achieving not only broadband absorption but also lightweight and high-strength multifunctionality.

In recent years, TPMS lattices have garnered widespread attention due to their exceptional mechanical properties. Leveraging their remarkable structural stability, Xiao et al. achieved enhanced structural energy absorption and damage resistance through a hierarchical design utilizing TPMS lattices. Additionally, in terms of wave-field regulation, TPMS lattices also demonstrate exceptional performance, aided by the thermal viscoelastic dissipation generated by their interconnected internal pores [19]. Zhang et al. designed a multi-layer sound-absorbing lattice structure, which achieved a maximum average sound absorption coefficient that is doubled compared to single-layer designs, with the maximum frequency increased by 127 times. In a compressed state, the elastic modulus and yield strength of the multi-layer structure are 0.43 times and 0.52 times that of the single-layer structure, respectively. This successfully realized the integration of structural acoustics and mechanical performance. Building upon this foundation, a further combination with sound-absorbing metamaterials was undertaken [20]. Tang et al. introduced an acoustic resonance effect into the structure by combining TPMS structures with micro-perforated panels, achieving a sound absorption coefficient greater than 0.5 at 900 Hz, with a maximum absorption coefficient exceeding 0.8. With the deepening of related research, researchers found that TPMS point arrays exhibit equally outstanding performance in electromagnetic wave absorption [21].

The interconnected porosity and triply orthogonal periodic gradients inherent in TPMS architectures significantly improve impedance matching and promote efficient electromagnetic energy dissipation via multi-scale scattering mechanisms, thereby exhibiting exceptional performance in the design of metamaterial absorbers. For example, Yu et al. proposed a novel polymer-derived silicon carbide (PDCsSiC)/silicon nitride TPMS structure by leveraging the interaction mechanisms between materials and structures. The porous TPMS structure effectively improves its impedance matching and increases the transmission path of electromagnetic waves, resulting in an effective absorption bandwidth (EAB) of 3.52 GHz at a thickness of 3.3 mm [22]. Subsequently, Xing et al. prepared ceramic-based materials that have been used for photopolymerization additive manufacturing, achieving excellent electromagnetic wave-absorption properties in the C, X, and Ku bands. Building on this, they designed and fabricated a new type of superstructure based on a P-type micro-curvature lattice, which realized an ultrawide effective absorption bandwidth of up to 11.36 GHz [23]. Qing et al. designed an ultra-wideband electromagnetic wave-absorbing element structure, inspired by the gyro structure in butterfly wings, which was prepared through additive manufacturing and dip-coating processes, successfully achieving excellent absorption performance in the frequency range of 2–40 GHz and exhibiting a stable wide-angle response [24].

**Figure 1:**
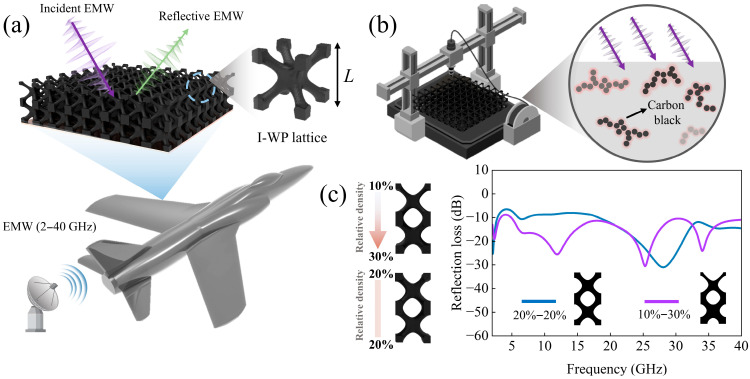
Schematic diagram of electromagnetic absorption metamaterial design method: (**a**) application requirements; (**b**) manufacture of test specimen; (**c**) verification of design results.

In summary, the TPMS lattices can effectively utilize their three-dimensional characteristics to further enhance their electromagnetic absorption properties while ensuring structural mechanical performance. Therefore, this study proposes a broadband microwave-absorbing lattice structure based on the I-WP TPMS, as shown in Figure 1a, which achieves effective radar stealth performance while maintaining lightweight characteristics and high mechanical strength. The I-WP lattice was efficiently fabricated through fused deposition modeling technology, as illustrated in Figure 1b, where the synergistic coupling between carbon black–polylactic acid composites and the architected geometry enables precise control over dielectric properties. By systematically analyzing the unit cell configurations, optimal absorption parameters were determined, followed by implementing a graded stacking strategy to broaden the operational bandwidth. Experimental validation using vector network analyzer measurements demonstrated strong consistency with the full-wave electromagnetic simulations.

## 2. Materials and Methods

### 2.1. Materials

The raw material is a Protopasta series conductive PLA from Protoplant (Vancouver, WA, USA). The PLA filaments contain 10 wt% carbon black with a diameter of 1.75 mm. The typical resistance of a 10 cm length of 1.75 mm filament is 2.0–3.5 kohm.

### 2.2. Main Printing Parameters

The test specimens were manufactured via a FDM 3D printing machine (JG AURORA, A6, Shenzhen, China). Printing was conducted at a temperature of 205 °C, with a 100% infill, at a speed of 30 mm/s, with a layer height of 0.15 mm, and by using a nozzle diameter of 0.2 mm. In addition, the printing process maintained a heated bed temperature of 60°.

### 2.3. Measurement of Electromagnetic Parameters

The composite filaments were fabricated into coaxial rings with an inner diameter of 3.04 mm, an outer diameter of 7.0 mm, and a thickness of 2 mm via FDM (fused deposition modeling) 3D printing. The instrument used for measuring the conductivity and magnetic permeability was the Ceyear 3671G network analyzer (Ceyear Technologies Co., Ltd., Qingdao, China). The frequency range was from 2 to 18 GHz.

## 3. Results and Discussion

### 3.1. EM-Absorbing Metamaterial Design

TPMS is a class of lattice structures with a zero mean curvature, featuring a low stress concentration and high design flexibility. The I-WP TPMS exhibits high symmetry and can be described by the following implicit function [25]:

(1) $$2\left[\cos\left(X\right)\cos\left(Y\right)+\cos\left(Z\right)\cos\left(Y\right)+\cos\left(X\right)\cos\left(Z\right)\right]-\left[\cos\left(2X\right)+\cos\left(2Y\right)+\cos\left(2Z\right)\right]=t,$$

where X, Y, and Z are rectangular Cartesian coordinates; and t is a level–set constant that controls the thickness of the I-WP TPMS.

The relative density ($\rho_{r}$) of the I-WP lattice structure that is considered in this work is defined as follows [26]:

(2) $$\rho_{r}=\frac{V_{s}}{V}=\frac{\rho_{s}}{\rho },$$

where *V* and ρ are the total volume and total density of the I-WP lattice structure, respectively, while $V_{S}$ and $\rho_{S}$ denote the solid volume and density of the base material. The relative density of the I-WP lattice structure is controlled by varying the thickness of the I-WP TPMS.

The high porosity and specific surface area of the structure not only facilitate a lightweight design but also enhance the interaction between electromagnetic waves and the structure. Meanwhile, the high shear modulus enables the structure to maintain its wave-absorbing performance under mechanical loading. Therefore, by combining dissipative materials with a lattice structure design, an effective regulation of the overall equivalent impedance can be achieved in subwavelength structures [1,18,27].

The relative density of the I-WP lattice structures exhibits a strong correlation with structural dimensions, significantly influencing both impedance-matching characteristics and lightweight design considerations. Researching the effect of a unit cell’s relative density on the wave-absorption performance of I-WP lattices is, therefore, crucial. This study examines the absorption properties of I-WP lattice unit cells (*L* = 10 mm) at various relative densities, establishing a foundation for subsequent design optimization.

The electromagnetic simulations were conducted using CST Studio Suite. Periodic boundary conditions were applied along the *X* and *Y* directions to simulate infinite array characteristics, while perfect electric conductor (PEC) and open boundary conditions were assigned to the *Z*+ and *Z*− planes, respectively.

**Figure 2:**
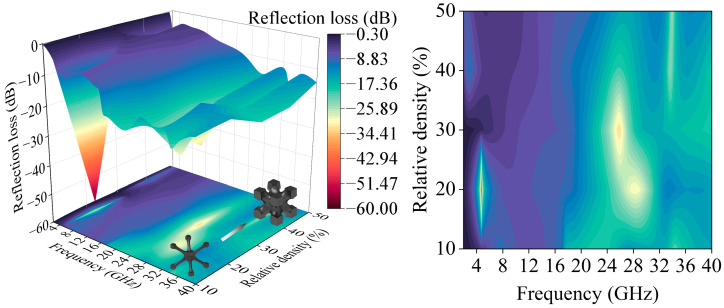
Influence of relative density on reflection loss of I-WP unit cell structure.

Simulations of unit cells with relative densities ranging from 10% to 50% revealed a non-monotonic absorption trend (Figure 2). A unit cell with a relative density of 20% exhibited a pronounced absorption peak between 2 and 20 GHz, demonstrating superior low-frequency performance. All five lattice structures, each with distinct relative densities, demonstrated over 90% wave absorption within the 20–40 GHz frequency range, thereby confirming their exceptional high-frequency absorption performance. Nevertheless, the unit cell with a relative density of 20% exhibited a restricted −10 dB bandwidth at lower frequencies, underscoring the necessity of thickness-optimized hierarchical designs to achieve broadband performance enhancement.

**Figure 3:**
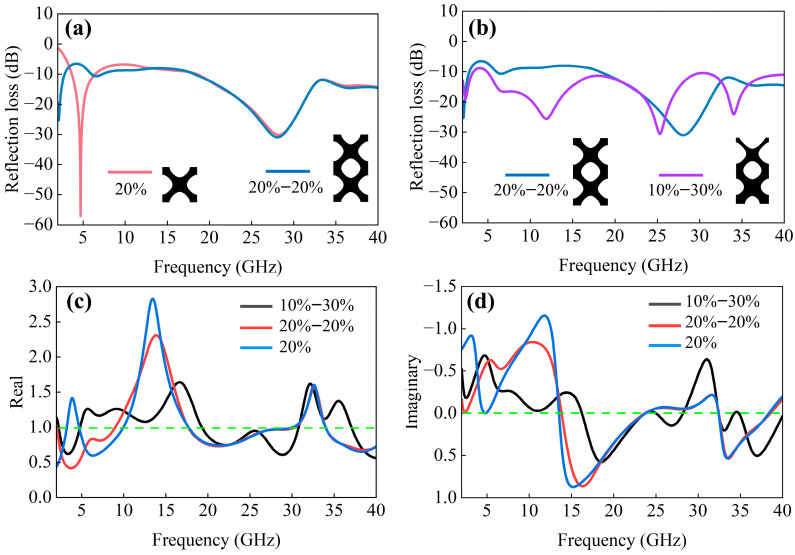
Reflection loss curve: (**a**) 20%–20% stack structure and 20% structure; (**b**) 20%–20% stack structure and 10–30% gradient structure. The real part (**c**) and the imaginary part (**d**) of the equivalent impedance are calculated based on the S-parameters.

Consequently, a double-layer stacked configuration with an average relative density of 20% was implemented, wherein each constituent single layer maintained an identical relative density of 20%, ensuring no variation in the overall mean density. This structure is referred to as “20–20%”. The simulation results, presented in Figure 3a, demonstrate that as the structural thickness increases, the absorption peak shifts toward lower frequencies while the low-frequency absorption performance is simultaneously enhanced. Nevertheless, increasing the structural thickness adversely affects the impedance-matching performance, thereby impeding electromagnetic wave penetration into the interior and limiting the full utilization of thickness-dependent dissipation mechanisms. The absorption curves of the stacked structure and the single-layer structure substantially overlap at high frequencies, and the absorption performance at high frequencies remains unaltered. Recent studies have demonstrated that gradient structural designs can enhance impedance-matching properties. For instance, Lim et al. developed a multi-layer gradient octet–truss structure, which exhibited significantly improved low-frequency electromagnetic wave absorption compared to conventional non-gradient multi-layer configurations [14]. The gradient structure enables sufficient electromagnetic wave dissipation along extended propagation paths. To achieve broadband absorption, a gradient structure with a relative density gradient was designed, featuring a continuous variation from 10% to 30% along the height direction while maintaining an overall average density of 20%. This structure is referred to as “10–30%”. As shown in Figure 3b, the 10–30% gradient structure demonstrates superior electromagnetic wave-absorption performance compared to the 20–20% structure. The gradual impedance gradient in the 10–30% structure effectively minimizes abrupt impedance discontinuities at the air–material interface, thereby enabling enhanced electromagnetic wave penetration depth. Furthermore, the 10–30% gradient structure exhibits enhanced absorption peak intensity and a broadened effective absorption bandwidth, and achieves a high reflection loss of less than −10 dB over a bandwidth exceeding 96% in the frequency range of 2–40 GHz.

Through the optimization design from a relative density and thickness to a gradient, the IWP–EMA achieved a wide frequency band for the absorption of electromagnetic waves. The optimization of relative density indicates that the unit cell with a relative density of 20% is the optimized result. However, the low-frequency absorption effect still needs to be improved. To improve low-frequency electromagnetic wave absorption while maintaining an average relative density of 20%, the bilayer stacking configuration was implemented to increase the overall structural thickness, thereby increasing the interaction distance between electromagnetic waves and the structure. It was found that simple stacking is not conducive to impedance matching. Therefore, in conjunction with relevant research in this field, a gradient design was implemented to address the impedance mismatch. The final optimized design results were obtained.

In principle, when the impedance of the structure is matched to the air (the real part of the impedance is 1, and the imaginary part is 0) to minimize the reflection, perfect absorption can be achieved [27]. The effective input impedance ($Z_{eff}$) of the IWP–EMA can be obtained from [28]:

(3) $$Z_{eff}=\sqrt{\frac{{\left(1+S_{11}\right)}^{2}-{S_{21}}^{2}}{{\left(1-S_{11}\right)}^{2}-{S_{21}}^{2}}}$$

where $S_{11}$ is the reflection coefficient, and $S_{21}$ is the transmission coefficient. Since the structure is backed by the PEC, $\left|S_{21}\right|$= 0. Based on the simulation of S-parameters, the real and imaginary parts of the impedance were calculated, resulting in three sets of data, which are plotted in Figure 3c,d. To quantify the effectiveness of impedance matching, the Mean Absolute Deviation (MAD) was calculated using Equation (4):

(4) $$MAD=\frac{1}{n}\sum_{i=1}^{n}\left|x_{i}-a\right|$$

where n is the number of data points in each group, $x_{i}$ is the value of the real part or the imaginary part, and a is the ideal value, which is 1 for the real part and 0 for the imaginary part. The calculated results are shown in Table 1. It can be seen that the 10–30% structure IWP–EMA is the smallest for both the MAD of the real part and the MAD of the imaginary part, indicating that this gradient IWP–EMA has a better impedance match with the air. Moreover, the real part of the impedance of this wave absorber is close to 1 at the four absorption peaks, while the imaginary part approaches 0.

### 3.2. Reflectance Test

**Figure 4:**
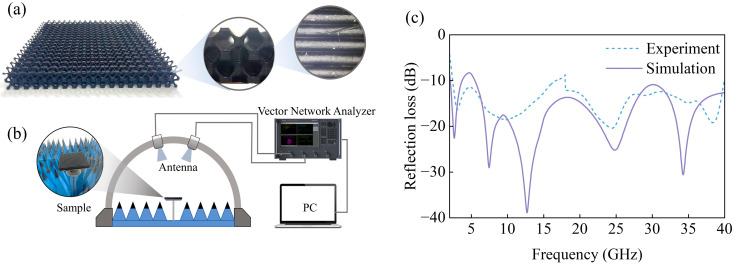
(**a**) Test specimen of IWP–EMA; (**b**) schematic of the arch method measurement configuration for reflection loss characterization; (**c**) comparison of the simulation and experimental reflection loss results.

Leveraging the self-supporting properties of the I-WP lattice, additive manufacturing was employed to fabricate a test specimen with high efficiency. The electromagnetic measurement specimen, with dimensions of 180 mm × 180 mm × 20 mm, consisted of an 18 × 18 array of gradient unit cells, as shown in Figure 4a.

The electromagnetic absorption performance was evaluated using the arch method. As shown in Figure 4b, transmit and receive broadband horn antennas were symmetrically arranged on a semicircular fixture, with the specimen positioned along the central axis. The antenna system covered dual frequency bands spanning 2–18 GHz and 18–40 GHz. Figure 4c compares the simulated and experimental results, revealing consistent absorption trends and peak alignment. Minor discrepancies arose from the FDM manufacturing artifacts, such as layer-induced surface roughness, along with inherent simulation and measurement uncertainties. Despite these variations, the design validity was conclusively demonstrated.

### 3.3. Energy Loss Mechanism

**Figure 5:**
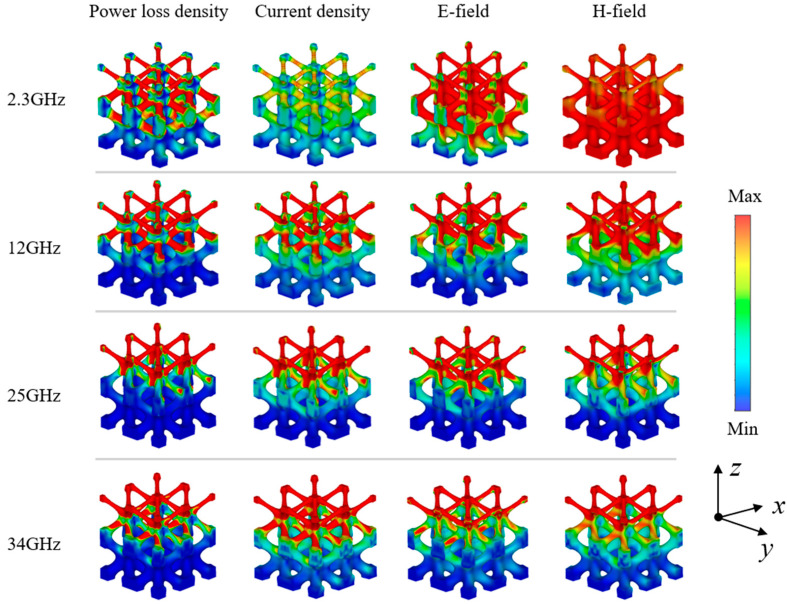
Surface field distribution of wave-absorbing structure.

**Figure 6:**
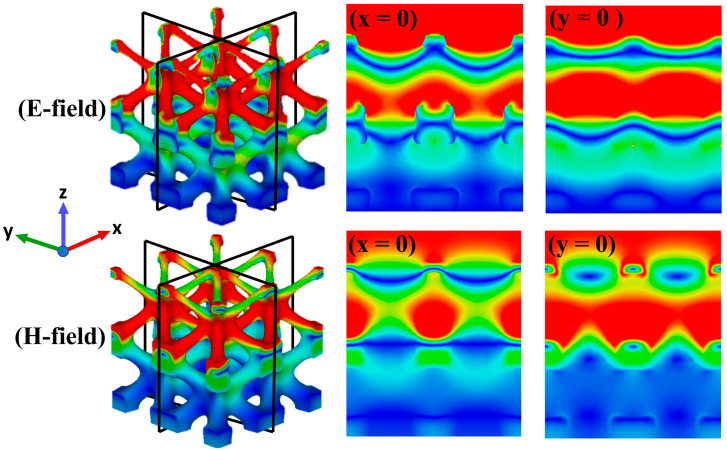
Distribution of electric and magnetic fields on a cross-section of an absorbing unit at 12 GHz under TE polarization.

From the experimental results, it can be seen that the I-WP structure produced four absorption peaks, located at 2.3 GHz, 12 GHz, 25 GHz, and 34 GHz. In order to analyze the energy loss characteristics of the lattice structure, we further simulated the power loss density, current density, electric field, and magnetic field distribution at the 2.3 GHz, 12 GHz, 25 GHz, and 34 GHz absorption peaks, as shown in Figure 5. For ease of analysis, the I-WP lattice structure is mainly divided into two parts: nodes and rods. The distribution of the electric field or the magnetic field shows the gradual dissipation of the incident electromagnetic wave in the structure. This indicates that the electromagnetic wave gradually attenuates as it traverses through the I-WP structure along its propagation direction. Figure 6 illustrates the distribution of the electric and magnetic fields in two mutually perpendicular cross-sections at 12 GHz under TE polarization. Alternating strong and weak stripes can be observed, indicating that the amplitude of the electric or magnetic field strength is enhanced in certain areas of space while weakening in others, which is attributed to the interference and superposition effects of the electromagnetic wave within the structure. Furthermore, it is observed that the electromagnetic wave deviates from its original linear propagation path upon reaching the edge of the structure, resulting in a diffraction phenomenon [27]. These phenomena indicate that complex interactions of electromagnetic waves occur within the structure [29], which together reduce transmission and reflection, increase the probability of electromagnetic waves entering the material interior, and thus improve the absorption capacity of IWP–EMA. The longitudinal comparative analysis shows that the power dissipation of the lattice structure is mainly distributed in the middle of the rod at low frequencies. At the same time, because of the strong penetration ability of low-frequency electromagnetic waves, they can reach the bottom of the structure and scatter at the edges and corners of the structure, thus increasing the propagation path of the microwave. With the increase of frequency, the number of microwaves reaching the bottom is less and less, and the power dissipation is also concentrated towards the slender rod on the top of the structure. Through further horizontal comparison and analysis, it can be seen that the distribution of current density and energy loss density is the same, indicating that the conductive network formed by carbon black dispersed in PLA provides a micro basis for the generation of a macro current, and a large amount of energy is dissipated in the form of ohmic heat energy [30,31,32]. In addition, the current formation also strengthens the interface polarization of dielectric loss materials, the micro-heterogeneous interface polarization and the nano-dipole polarization of the precipitation phase, and enhances the dissipative ability of the structure.

### 3.4. Angle-Sensitive Properties

**Figure 7:**
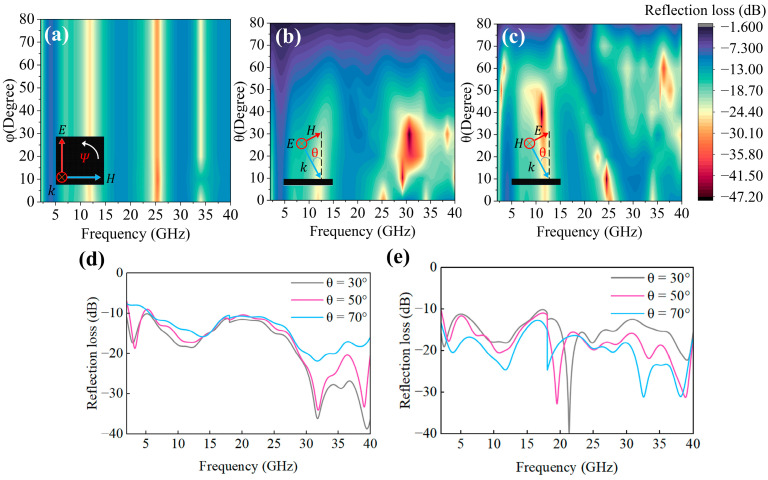
Angular sensitivity: (**a**) Reflection loss of the IWP–EMA for different polarization angles (φ) under normal incidence; reflection loss of the IWP–EMA under oblique incidence for the TE mode (**b**) and TM mode (**c**). Experiments on the incidence at different angles θ: (**d**) TE mode and (**e**) TM mode.

**Figure 8:**
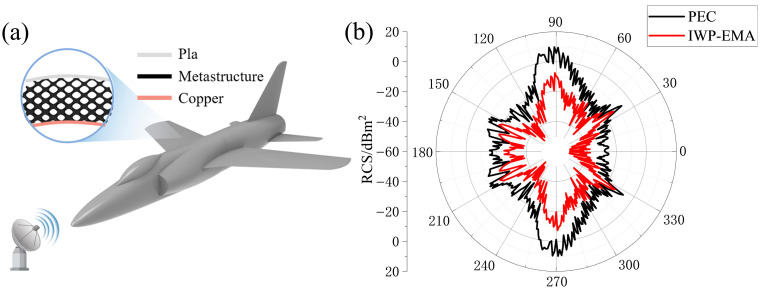
(**a**) UAV with IWP–EMA; (**b**) under TE polarization, the circumferential RCS curves of the UAV are at 12 GHz.

In practical applications, the absorbing properties of absorbing materials are related to polarization and the incidence angle, and isotropic superstructures show superior insensitive polarization [33]. Therefore, the absorption characteristics of TE and TM polarization at different incident angles are simulated. φ is the azimuth, the angle at which the plane of incidence (the plane formed by the direction of the propagation of the incident wave and the normal surface area of the material) rotates around the normal area within the surface plane of the material. Changing the φ angle will not affect the absorption characteristics of the electromagnetic waves, basically, as shown in Figure 7a, which has excellent angle sensitivity. θ is the angle of incidence—the angle between the direction of the propagation of the incident electromagnetic wave and the direction of the normal area of the surface of the material. The reflectivity of electromagnetic waves at different incident angles θ under TE polarization is shown in Figure 7b. It can be seen that the wave-absorption performance in the TE mode is stable to 50°. The reflectivity of electromagnetic waves at different incident angles θ under TM polarization is shown in Figure 7c. It can be seen that the wave-absorption performance in the TM mode is stable to 70°. In order to further verify the angular dependence of the absorption characteristics, three different theta angle experiments were carried out in the TE mode and TM mode by using the arch method. As shown in Figure 8, the angles of incidence at 30°, 50°, and 70° exhibit excellent broadband absorption characteristics in both the TE and TM modes, with the experimental results providing effective validation of the simulations. Furthermore, the simulation results under TE polarization can stabilize the incident angle to 50°, while the experimental results can stabilize the incident angle to 70°. Therefore, 70° is considered the angle that can be reliably achieved under TE polarization. In the TE mode, four main absorption peaks were observed; within the frequency range of 5–40 GHz, as the angle of incidence increased, the positions of the three absorption peaks remained relatively unchanged, while their intensities gradually decreased, all being below –10 dB. In the TM mode, five main absorption peaks were observed; within the same frequency range, as the angle of incidence increased, the positions of the absorption peaks shifted forward, and the intensity of the absorption peaks exhibited no clear pattern, but all remained below –10 dB. Under TM polarization, the metamaterials show better angle sensitivity because the magnetic field component is parallel to the surface under TM polarization, and the electric field component changes with the incident angle. Therefore, the increase in the electric field action distance excites more current, which increases the loss of electromagnetic waves. Under TE polarization, the electric field component is parallel to the surface, while the magnetic field component decreases with an increase in the incidence angle, which weakens the magnetic resonance and reduces the absorption [16].

### 3.5. Application Demonstration

In order to visually display the wave-absorption performance of this metamaterial, as shown in Figure 8a, a UAV model with dimensions of 2 × 1.4 × 1.5 m was selected for the single-station radar RCS simulation, and the simulation frequency was set at 12 GHz. RCS is a physical quantity that measures the ability of a target to reflect electromagnetic waves under radar illumination. The larger the RCS, the stronger the reflected electromagnetic waves, making it easier for the target to be detected on radar displays. Single-station RCS can be described as [16]:

(5) $$\sigma =\underset{R\to \infty }{lim}\;4\pi R^{2}\frac{{\left|E_{S}\right|}^{2}}{{\left|E_{i}\right|}^{2}}=\underset{R\to \infty }{lim}\;4\pi R^{2}\frac{{\left|H_{S}\right|}^{2}}{{\left|H_{i}\right|}^{2}}$$

where R is the distance from the target to the radar antenna, $E_{S}$ and $H_{S}$ represent the electromagnetic field intensity of the target’s scattering field at the radar, while $E_{i}$ and $H_{i}$ represent the electromagnetic field intensity of the incident radar wave at the target, respectively.

As shown in Figure 8b, compared with the model coating PEC, the circumferential RCS curve of the model with this type of metamaterial on its surface in the forward and backward directions is significantly reduced, and the average RCS is reduced from −16.83 dBm^2^ to −30.13 dBm^2^, which proves the prospect of the I-WP lattice structure in electromagnetic stealth applications. Considering the aerodynamics, a thin layer of electromagnetic wave permeability can be attached to the surface of the IWP–EMA to reduce the impact of holes on aerodynamic performance.

## 4. Conclusions

This paper proposes an electromagnetic wave-absorbing structure based on I-WP, which achieves a high reflection loss of less than −10dB over a bandwidth exceeding 96% in the frequency range of 2–40GHz, aided by efficient impedance characteristic control from the gradient design and various macro/micro energy dissipation mechanisms. Furthermore, the three-dimensional characteristics of this metamaterial provide excellent insensitivity to the angle of incidence, exhibiting stable frequency responses under both TE and TM polarizations within a 70° angle of incidence range. Additionally, by applying the designed metamaterials to a UAV model, the average RCS was reduced from −16.83 dBm^2^ to −30.13 dBm^2^, verifying its effectiveness in reducing the average radar cross-section. Such ultra-wideband, wide-angle electromagnetic wave-absorbing metamaterials hold significant application prospects across various fields, including aerospace, military, and communications, and also offer insights for the electromagnetic absorption design of three-dimensional lattice structures.

## Figures and Tables

**Table 1:** The MAD calculation results.

Structure	Re(MAD)	Im(MAD)
20%	0.3208	0.3900
20–20%	0.3182	0.3539
10–30%	0.2426	0.2488

## Data Availability

The original contributions presented in this study are included in the article. Further inquiries can be directed to the corresponding authors.
